# Early-life homeostatic differentiation of thymus-resident B cells into memory B cells

**DOI:** 10.3389/fimmu.2025.1567788

**Published:** 2025-03-28

**Authors:** Justine Castañeda, Lilian Poblete, Mariana V. Rosemblatt, Daniela Sauma, Mario Rosemblatt, María Rosa Bono, Sarah Nuñez

**Affiliations:** 1 Escuela de Postgrado, Facultad de Medicina, Universidad de Chile, Santiago, Chile; 2 Departamento de Biología, Facultad de Ciencias, Universidad de Chile, Santiago, Chile; 3 Facultad de Medicina y Ciencia, Universidad San Sebastián, Sede Los Leones, Santiago, Chile; 4 Centro Ciencia & Vida, Santiago, Chile

**Keywords:** thymic B cells, memory B cell differentiation, Ig class-switching, T cell development, negative selection

## Abstract

The thymus contains various antigen-presenting cells, including B cells, which remain activated even under steady-state conditions, suggesting ongoing local stimulation. In this study, we identify class-switched memory B cells in the thymus. Some of these cells switch their immunoglobulin to IgG2b and IgA, and express typical memory markers CD73 and PD-L2. Memory B cell differentiation in the thymus begins in neonatal mice, preceding the appearance of class-switched B cells in other peripheral lymphoid organs. Notably, exposure to environmental antigens does not influence their differentiation. Additionally, cognate interaction with CD4^+^ positive thymocytes is crucial for the development of memory B cells in the thymus. Our findings demonstrate that the thymus supports the local differentiation of memory B cells through a steady-state process, independent of foreign antigen stimulation and driven by interactions with developing T cells.

## Introduction

During their development, immature T cells engage several types of antigen-presenting cells (APCs) to ensure the selection of a functional, self-tolerant repertoire of mature T cells (1–7). In addition to medullary epithelial cells (mTEC) and dendritic cells, the thymic medulla houses a significant subset of B cells (1–8). Thymic B cells play a non-redundant role in central tolerance and act as specialized APCs that mediate clonal deletion and promote the differentiation of thymic Tregs (9, 10). Under steady-state conditions they exhibit several hallmarks of activated B cells, including high expression of MHC-II and significantly higher levels of co-stimulatory molecules CD80 and CD86 compared to resting B cells in the spleen (7, 11–14). A fraction of thymic B cells expresses the AIRE transcription factor, which is known to enable the promiscuous expression of self-antigens in mTEC (11, 12). Interestingly, previous studies have noted that some thymic B cells have undergone class-switching, however, the nature and origin of thymic class-switched B cells has not been investigated.

In a specific humoral response, naive B cells are activated and begin their differentiation process in secondary lymphoid organs upon encountering cognate antigens. In this process, B cells engage cognate CD4^+^ T cells and receive signals to differentiate into either memory B cells or plasma cells. These specialized cells can respond more rapidly and robustly upon subsequent encounters with their specific antigen (15, 16). Studying memory B cells in mice has been challenging due to the lack of specific markers that distinguish them. Traditionally, they have been identified as IgD^-^IgM^+^ i.e., IgM memory, or IgD^-^IgM^-^ i.e., switched memory cells that lack the expression of germinal center markers (17, 18). Recently, new markers such as CD73, PD-L2, and CD80, combined with the expression of IgD and IgM, have allowed for better identification of memory B cell subsets (19–24). Memory B cells are found in the blood and reside in various lymphoid and non-lymphoid tissues (22, 25–30).

In this work, we show that without external antigenic stimulation thymic B cells undergo class-switching and exhibit phenotypic features akin to conventional memory cells. This process was not affected by a reduction of peripheral memory formation in mice treated with perinatal antibiotics, suggesting an activation mechanism that is independent of foreign antigen recognition. Additionally, class-switched B cells could be detected in the thymus of neonatal mice, prior to their appearance in other secondary lymphoid organs. In the absence of effective B-T interaction in OT-II mice, memory B cell differentiation was specifically reduced in the thymus. Together, these findings demonstrate that the thymus supports memory B cell differentiation through an unconventional pathway, independent of external antigen exposure, via interaction with developing T cells.

## Materials and methods

### Mice

C57BL/6 (B6 CD45.2), B6.SJL-*Ptpcr^a^Ptpcr^b^/*BoyJ (B6.SJL CD45.1), BALBcj, and OT-II (B6.Cg-Tg (TcraTcb)425Cbn/J) mice were purchased from the Jackson Laboratory (Bar Harbor, ME, USA). Unless otherwise specified, mice aged 6 to 12 weeks were not selected based on sex, except for the parabiosis experiment, which was conducted exclusively with female mice. For the study design, appropriate sample sizes were estimated using the method of Charan and Kantharia (31), with Power of 80% and Type 1 error of 5%. Anticipated effect size and standard deviation was extracted from previous analyses of thymic B cells (32). For perinatal antibiotic experiments, the estimated sample size was increased due to potential effects of antibiotic treatment on litter size and viability. All mice were maintained at the animal facility of Centro Científico y Tecnológico de Excelencia Ciencia & Vida, Santiago, Chile. Mice were kept in a conventional animal facility on a standard diet, at 20-23°C, and a 12-hour light/dark cycle, except for the perinatal antibiotic group, which were housed in cages with HEPA-filtered ventilation systems, with autoclaved water and food to minimized exposure to environmental antigens. Mice were euthanized via carbon dioxide (CO_2_) inhalation. Neonates were deeply anesthetized with Sevoflurane, followed by quick decapitation to ensure death. All animal procedures were conducted under the institutional regulations of Fundación Ciencia & Vida and approved by the ethical review committee of Centro Ciencia & Vida.

### Cell isolation

Splenocytes were obtained by organ perfusion with RPMI + 10% FBS medium to generate a cell suspension. Red blood cells were eliminated by resuspending cells in 2mL of red blood lysis buffer (RBC lysis buffer, BioLegend) for 5 minutes at 4°C. To obtain cell suspensions of the thymus and mesenteric lymph nodes, both organs were extracted and mechanically dissociated using a manual tissue homogenizer. The resulting cell suspension was filtered through a 90 μm mesh. To isolate cells from Peyer´s patches, the small intestine was extracted and placed in a petri dish with cold PBS. Peyer´s patches (6-8 per intestine) were dissected along the length of small intestine and mechanically dissociated using a manual tissue homogenizer. The resulting cell suspension was filtered through a 90 μm mesh. In experiments with neonatal mice, collection of the spleen, thymus and mesenteric lymph nodes was aided with a dissection microscope.

### Flow cytometry

To analyze a representative number of B cells, the following cells numbers were used from each organ: 2,5x10^6^ spleen cells, 2,5x10^6^ mesenteric lymph nodes cells, 2,5x10^6^ Peyer´s patches cells, and 5x10^6^ thymic cells. Cells were incubated for 15 min at 4°C with Fcγ-R blocking antibody (CD16/32) to reduce non-specific binding. The cells were then surface-stained for 25 minutes at 4°C in the dark in FACS buffer (PBS containing 2% fetal bovine serum) with the following mouse monoclonal antibodies: GL-7 AF488 (GL7, BioLegend), PD-L2 PE (TY25, BioLegend), CD73 APC (TY/11.8, BioLegend), CD21/35 BV421 (7Ea, BioLegend), B220 BV510 (RA3-6B2, BioLegend), IgD BV650 (11-2bc.2a, BioLegend), CD19 BV711 (6D5, BioLegend), IgG2b FITC (RMG2b-1, BioLegend), IgM PE Cy7 (RMM-1, BioLegend), CD45.1 PE Cy7 (A20, BioLegend), CD45.2 APC (104, BioLegend), GL-7 AF647 (GL7, BioLegend) and IgA PE (mA-6E1, Invitrogen). Cells were washed and resuspended in FACS buffer for acquisition. Propidium iodide (PI) was used to discriminate between live and dead cells. To rule out a potential contamination of blood B cells in the immunophenotype analysis of thymic B cells, we separately performed intravascular labelling of CD45^+^ leucocytes combined with surface staining (**Supplementary Figure 1B**). The cells were analyzed on a FACSAria III analyzer (BD Biosciences) and Aurora 5L analyzer (Cytek). All data were processed using FlowJo version 10.4 (Tree Star, Inc.) software.

### t-SNE and cluster analysis

For t-SNE visualization of memory B cells in the thymus, spleen, and Peyer’s Patches, a multicolor flow cytometry panel was used, including 12 parameters (FSC, SSC, Viability, CD21-35, B220, IgD, CD19, CD23, PD-L2, CD73, GL-7, and CD43). Cells were compensated for spillover between channels and pre-gated on CD45^+^B220^+^CD19^+^ live B cell singlets using FlowJo. FlowJo workspace was imported into the R environment using CytoML, FlowWorkspace, and FlowCore packages. The intensity values of markers expression were the biexp-transformed via flowjo_biexp_trans function of FlowWorkspace using parameters ChannelRange = 4096, maxValue = 262144, pos = 4.5, neg = 0, and with Basis = 10. Subsequently, 10.000 cell events from each mouse were randomly selected and combined for 30.000 single cells. Sample data was subjected to dimensionality reduction by t-Distributed Stochastic Neighbor Embedding (tSNE) using the RtSNE package. Seven parameters were utilized for tSNE construction (CD21-35, IgD, CD23, PD-L2, CD73, GL-7, and CD43), and the parameters were set to interactions = 3000 and perplexity = 30. After dimensionality reduction, automatic clustering was performed using the DBSCAN package.

### Principal component analysis

PCA of memory B cells from thymus, spleen, and Peyer’s patches was performed using the prcomp package in R studio. PCA-transformed data with similar profiles are grouped using fviz_pca_ind. To infer clusters from PCA-transformed data according to variables, we use the fviz_pca_biplot package.

### Parabiosis surgery

Parabiosis surgery was performed as described (33), using pairs of female mice cohoused for two weeks before surgery. Mice were anesthetized intraperitoneally with Avertin (2,2,2-Tribromoethanol, Sigma Aldrich) at 250mg/kg. The surgical area was shaved, and a longitudinal incision was made in the skin from the elbow to the knee. The skin was loosened from the connective tissue to leave approximately 1cm of free skin. The mice were joined with non-absorbable sutures in the elbow and knee joints, and the skin was closed with stainless steel wound clips. Body temperature was maintained using a heating pad during surgery and recovery. For analgesia, mice were given Tramadol at a dose of 50mg/kg subcutaneously every 12 hours prior to and for the following 72 hours after surgery.

### Perinatal antibiotics treatment

We performed perinatal antibiotics treatment as described previously to establish a model with reduced colonization by commensal microbiota (34–37). Pregnant mice were given a cocktail of antibiotics dissolved in water, including Ampicillin (1mg/mL, US Biological), Vancomycin (0,5mg/mL, PhytoTech Lab), Neomycin (1mg/mL, PhytoTech Lab), and Metronidazole (1mg/mL, Cayman Chemical), at least 7 days before the expected date of birth. The pups were then maintained with the same antibiotics cocktail for 8 weeks until they were euthanized. The antibiotics solution was kept protected from light and replaced twice per week.

### Bacteria quantification

Before euthanizing control and antibiotic-treated mice, stool samples were collected for bacteria quantification using Syto™ BC green fluorescent nucleic acid stain (Invitrogen) according to the manufacturer’s instructions. Cell counts were obtained by adding Precision Count Bead (Biolegend) and analyzing the sample in a FACSCanto II (BD, Bioscience). Dead bacteria were excluded from the analysis using Propidium iodide.

### Peptide injection

Two-month-old OT-II mice were injected intravenously with 85nmol of pOVA (OVA_323-339_, ISQAVHAAHAEINEAGR) as described in (38). One week after the injection, mice were euthanized.

### Statistical analysis

GraphPad Prism version 9.0.1 software was used for statistical analysis. The normality of all data was assessed using the Shapiro-Wilk test. Unpaired t-test, Mann-Whitney t-test, one-way ANOVA with Tukey post-test, Kruskall-Wallis with Dunn’s post-test, and correlation test were used for data analysis and the generation of p or r^2^ value. The exact p-values that were statistically significant are reported in the graphs.

## Results

### Thymus-resident class-switched memory B cells under steady-state conditions

**Figure 1:**
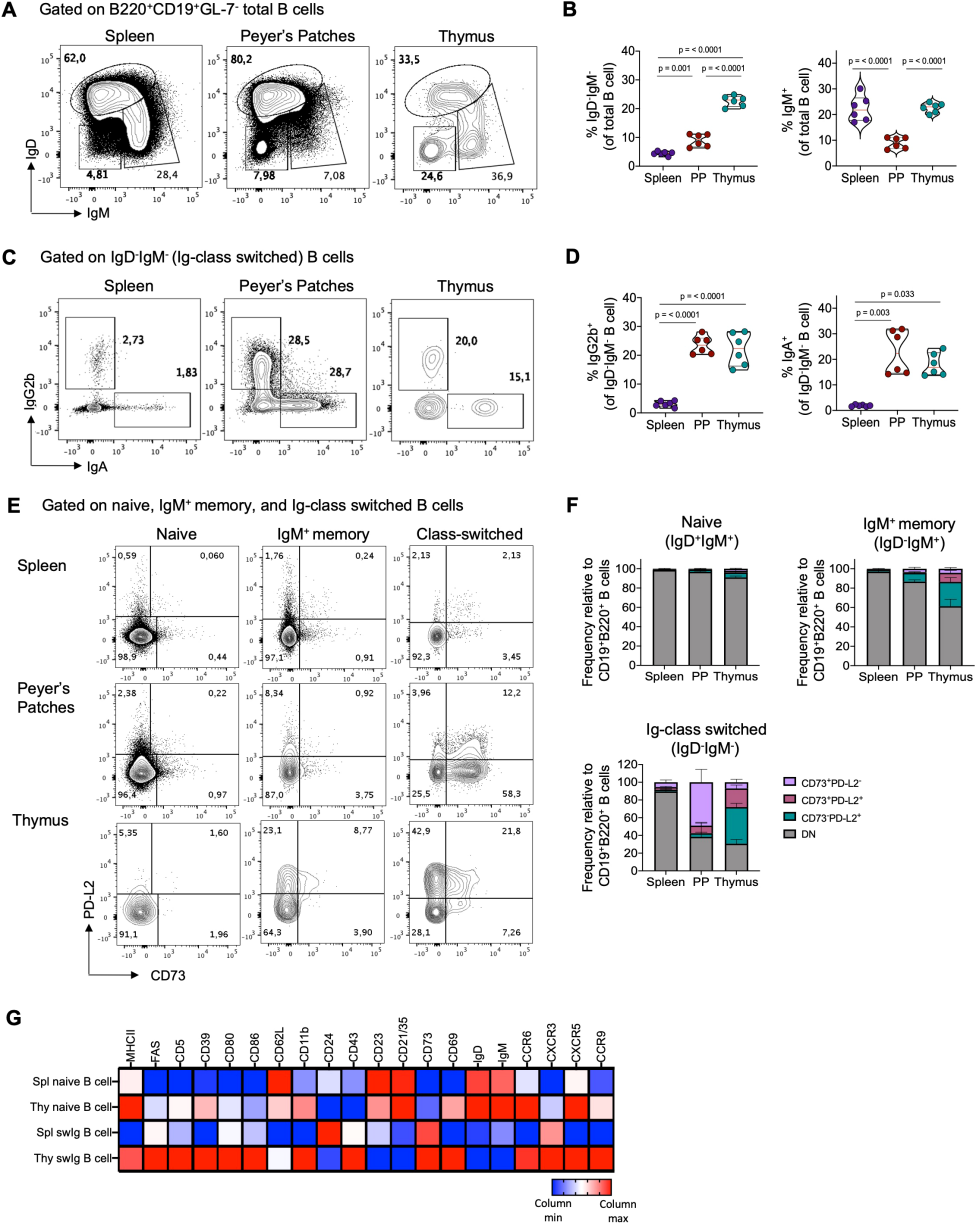
Thymic B cells exhibit high expression of markers associated with a memory phenotype under steady-state condition. **(A)** Representative dot plots of naïve (IgD^+^IgM^+^), Ig-class-switched (IgD^-^IgM^-^), and IgM^+^ memory (IgD^-^IgM^+^) B cells within the B220^+^CD19^+^GL-7^-^ gate in the spleen, Peyer´s patches, and thymus of 3-month-old C57BL/6 mice (n = 6). **(B)** Percentage of total Ig-class-switched and IgM^+^ memory B cells gated as in **(A)**. **(C)** Representative dot plots of class-switched IgG2b and IgA cells gated on IgD^-^IgM^-^ B cells. **(D)** Percentage of IgG2b^+^ and IgA^+^ isotypes among IgD^-^IgM^-^ gated B cells from the spleen, Peyer´s patches, and thymus (n = 6). **(E)** Dot plot of CD73 and PD-L2 expression in naïve (IgD^+^IgM^+^), IgM^+^ memory (IgD^-^IgM^+^), and Ig-class-switched (IgD^-^IgM^-^) B cells subsets in the spleen, Peyer´s patches, and thymus (n = 6). **(F)** Proportion of CD73^+^PD-L2^-^, CD73^+^PD-L2^+^, and CD73^-^PD-L2^+^ within naïve, IgM^+^ memory, and Ig class-switched B cells gated as in **(E)**. **(G)** Heat map displaying mean fluorescence intensities (MFI) of indicated markers in naïve and Ig-class switched (swIg) B cells in the spleen (spl) and thymus (thy) measured by flow cytometry. Data were log2-transformed and visualized as relative expression per column (raw and log2-transformed MFI data is presented in **Supplementary Table 1**). Representative gating strategy for the analysis of B cells and memory subsets is shown in **Supplementary Figure 1A**. Each dot represents an individual mouse. Data was analyzed using one-way ANOVA with Tukey´s post-test for multiple comparisons **(A–C)**, except for IgA^+^ B cells, which were analyzed using the Kruskal-Wallis with Dunn´s post-test for multiple comparisons. PP, Peyer’s patches.

Mature CD19^+^B220^+^ B cells constitute approximately 0.1-0.5% of total thymic cells (1–7). IgD and IgM expression analysis in thymic B cells reveals heterogeneity, with around 20-25% being class-switched IgD^-^IgM^-^ cells and 20% being IgD^-^IgM^+^ cells (**Figures 1A, B**). Interestingly, the proportion of Ig-switched cells is notably higher in the thymus than in other lymphoid organs, such as Peyer’s patches (PP), where B cells are continuously activated by commensal and dietary antigens (39, 40). Furthermore, thymic B cells primarily undergo isotype switching to IgG2b and IgA, with lesser extents to IgG1 and IgG2ac (**Figures 1C, D**, **Supplementary Figures 2A, B**).

Our investigation explored whether thymic class-switched B cells exhibit phenotypic similarities to conventional memory B cells found in peripheral lymphoid organs. We assessed the expression of CD73 and PD-L2, markers associated with the maturity and functional potential of antigen-specific memory B cells (17, 19). While the majority (>90%) of naïve B cells in the spleen, Peyer’s patches, and thymus lacked CD73 and PD-L2 expression, class-switched B cells in the thymus were predominantly PD-L2^+^ or CD73^+^PD-L2^+^, whereas those in PP were mostly CD73^+^ (**Figures 1E, F**). Additionally, compared to splenic class-switched B cells and naïve B cells, thymic class-switched B cells exhibited higher expression levels of activation molecules such as MHC-II, CD80, CD86, CD69, CXCR3, and CXCR5. They also displayed elevated expression of CD39 and CD43, which are highly expressed memory markers in mouse and human memory B cells (41) (**Figure 1G**). Interestingly, while all B cell populations in the analyzed organs shared signature expression of memory markers, principal component analysis (PCA) and dimensionality reduction analysis (t-SNE) revealed differences between thymic memory B cell subsets and those in the spleen and PP, possibly reflecting their distinct localization and function within the thymus (**Supplementary Figures 2C-E**).

Our examination of BALB/c mice confirmed that the properties of thymic B cells can be generalized independently of mouse strain. Similarly to B6 mice, the thymus of BALB/c mice contained a comparable proportion of Ig-switched, IgM^+^ memory cells, IgG2b^+^, and IgA^+^ B cells, and were predominantly PD-L2^+^ and CD73^+^PD-L2^+^ (**Supplementary Figure 3**). These findings underscore that the thymus houses a significant population of memory B cells under steady-state condition that is phenotypically distinct from memory B cells in secondary lymphoid organs.

### Thymic memory B cells are tissue-resident and differentiate intrathymically

**Figure 2:**
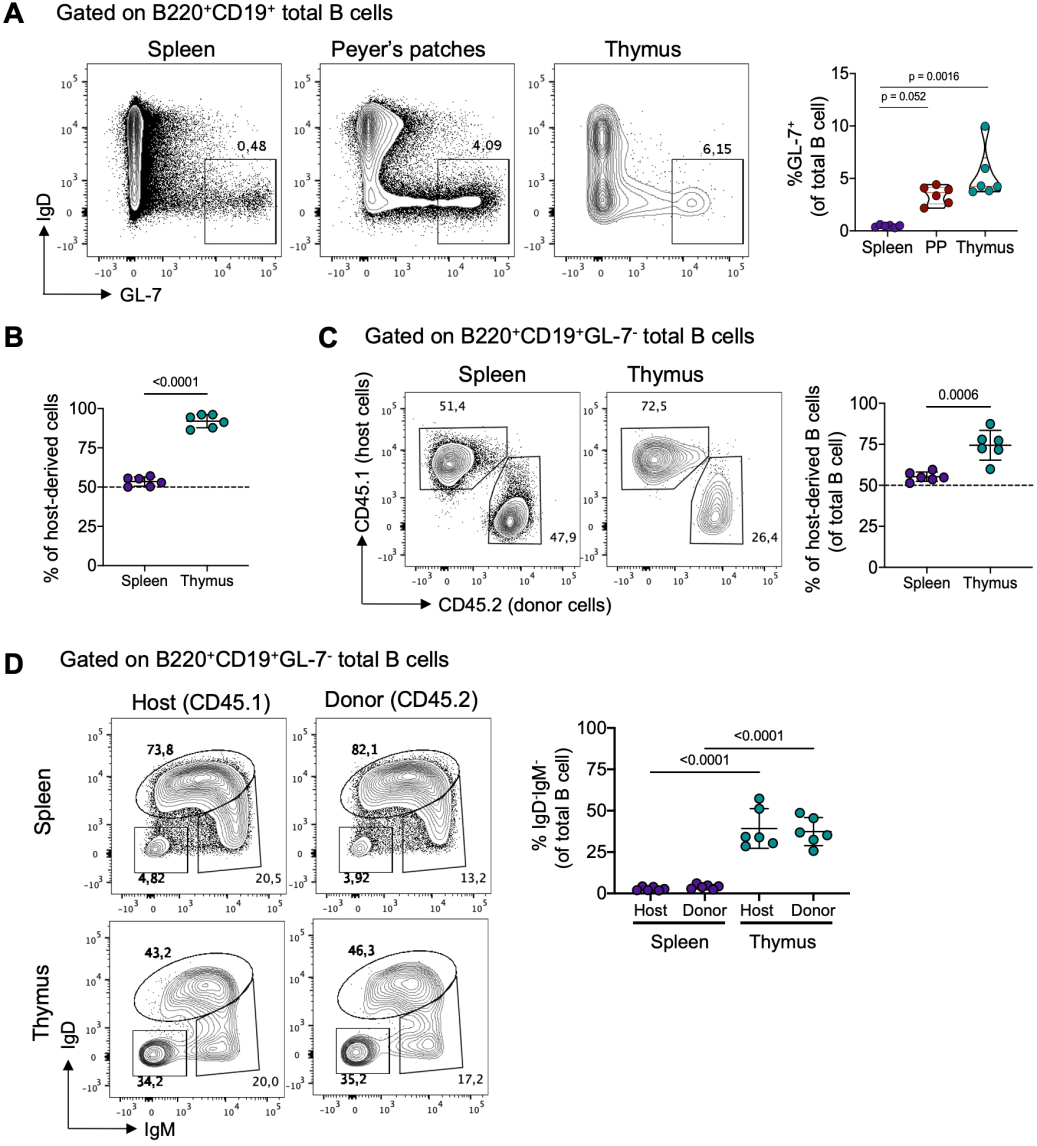
Local differentiation and tissue-residency of thymic memory B cells. **(A)** Representative dot plots (left) of germinal center B cells (IgD^-^GL-7^+^) and the frequency (right) of IgD^-^GL-7^+^ B cells in the spleen, Peyer’s patches, and thymus of from 3-month-old C57BL/6 mice (n = 6). **(B)** Frequency of total host-derived cells in splenic and thymic B cells from 3-month-old C57BL/6 parabiotic mice. Host-derived cells were identified using CD45.1 and CD45.2 antibodies. **(C)** Representative dot plots (left) and frequency (right) of host-derived B cells in splenic and thymic B cells from parabiotic mice (n = 6). **(D)** Representative dot plots and frequency of splenic (upper) and thymic (lower) class-switched B cells (IgD^-^IgM^-^) from host-derived (CD45.1) and donor-derived (CD45.2) mice (n = 6). Each dot represents an individual mouse. Data were analyzed using the Kruskal-Wallis test with Dunn’s post-test for multiple comparisons **(A)**, unpaired t-test **(C)**, and one-way ANOVA with Tukey’s *post-hoc* test for multiple comparisons. PP, Peyer’s patches.

During memory B cells and plasma cell formation, activated B cells either differentiate rapidly through an extrafollicular response or migrate into germinal centers (GC) in secondary lymphoid organs where they increase their affinity for antigens and differentiate into memory or plasma cells (26). To investigate whether thymic B cell activation and differentiation occurs locally, we examined GL-7 expression, a marker for activated B cells in the extrafollicular and germinal center response. Interestingly, we found GL-7^+^ B cells in the spleen, Peyer’s patches, and the thymus, with thymic B cells displaying the highest proportion of GL-7^+^ cells, similar to Peyer’s patches (**Figure 2A**). This observation aligns with our previous finding, suggesting a significant basal differentiation into memory B cells in the thymus.

To confirm that thymic memory B cells are primarily derived from local differentiation rather than migrating from the periphery, we generated parabiotic B6 CD45.1 and CD45.2 congenic mice. In parabionts joined for 6 weeks, splenic cells exchanged readily, reaching an equilibrium of about 50% of host and donor cells. In contrast, minimal entry of circulating cells into the thymus was observed, with the majority of CD4^+^CD8^+^ thymocytes (>90%) derived from the host (**Figure 2B**; **Supplementary Figure 4A**). Furthermore, the proportion of donor-derived B cells in the spleen reached equilibrium with donor B cells. In contrast, only approximately 25% of thymic B cells originated from the donor partner, suggesting a tissue-resident bias (**Figure 2C**). Importantly, among the donor-derived B cells, class-switched cells remained minimal in the spleen (<5%), whereas in the thymus, around 30% acquired a class-switched phenotype (IgD^-^IgM^-^), resembling the proportion of endogenous thymic B cells (**Figure 2D**). Thymic B cell subsets predominantly consisted of host-derived cells, while splenic B cell subsets showed an equal proportion of host and donor-derived cells (**Supplementary Figure 4B**). These findings collectively show that thymic memory B cells differentiate locally and remain as a tissue-resident population.

### Detection of class-switched B cells in the neonatal thymus

**Figure 3:**
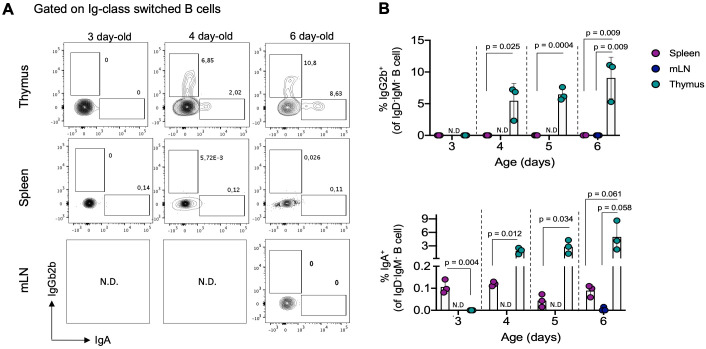
Thymic IgG2b^+^ and IgA^+^ B cells are detectable in the first days of life. **(A)** Representative dot plots of IgG2b^+^ and IgA^+^ B cells within the IgD^-^IgM^-^ gate in the thymus, spleen, and mesenteric lymph nodes in neonatal mice aged 3, 4, and 6 days (n = 3 for each age group). **(B)** Percentage of IgG2b^+^ (top) and IgA^+^ (bottom) B cells in analyzed organs from neonatal mice, as gated in **(A)**. Each dot on the graph represents an individual mouse of the indicated age. Data are presented as mean ± standard deviation (SD). Comparisons were analyzed using multiple unpaired t-tests analyzed data for comparisons. mLN, mesenteric lymph nodes; N.D, no determined.

Given our results showing intrathymic differentiation of memory B cells under steady-state conditions, we investigated whether the development of thymic memory B cells begins early stage in life, preceding the emergence of adaptive responses in the periphery. To address this, we tracked IgG2b^+^ and IgA^+^ populations (the most represented Ig classes in the thymus) in the thymus, spleen, and mesenteric lymph node (mLN) of neonatal mice (3 to 6-day-old). Both populations of memory B cells were virtually undetectable in the spleen and mLN of neonatal mice. Interestingly, we observed that IgG2b^+^ and IgA^+^ B cells became detectable in the thymus starting from 4 days after birth, with significantly higher frequencies compared to B cells from the spleen and mLN during the neonatal stage (**Figure 3**). This early development of memory B cells in the thymus, even before their detection in secondary lymphoid organs, suggests that differentiation into memory B cells in the thymus is a process independent from conventional peripheral memory formation.

### Distinct influence of environmental antigens on thymic and peripheral memory B cell development

**Figure 4:**
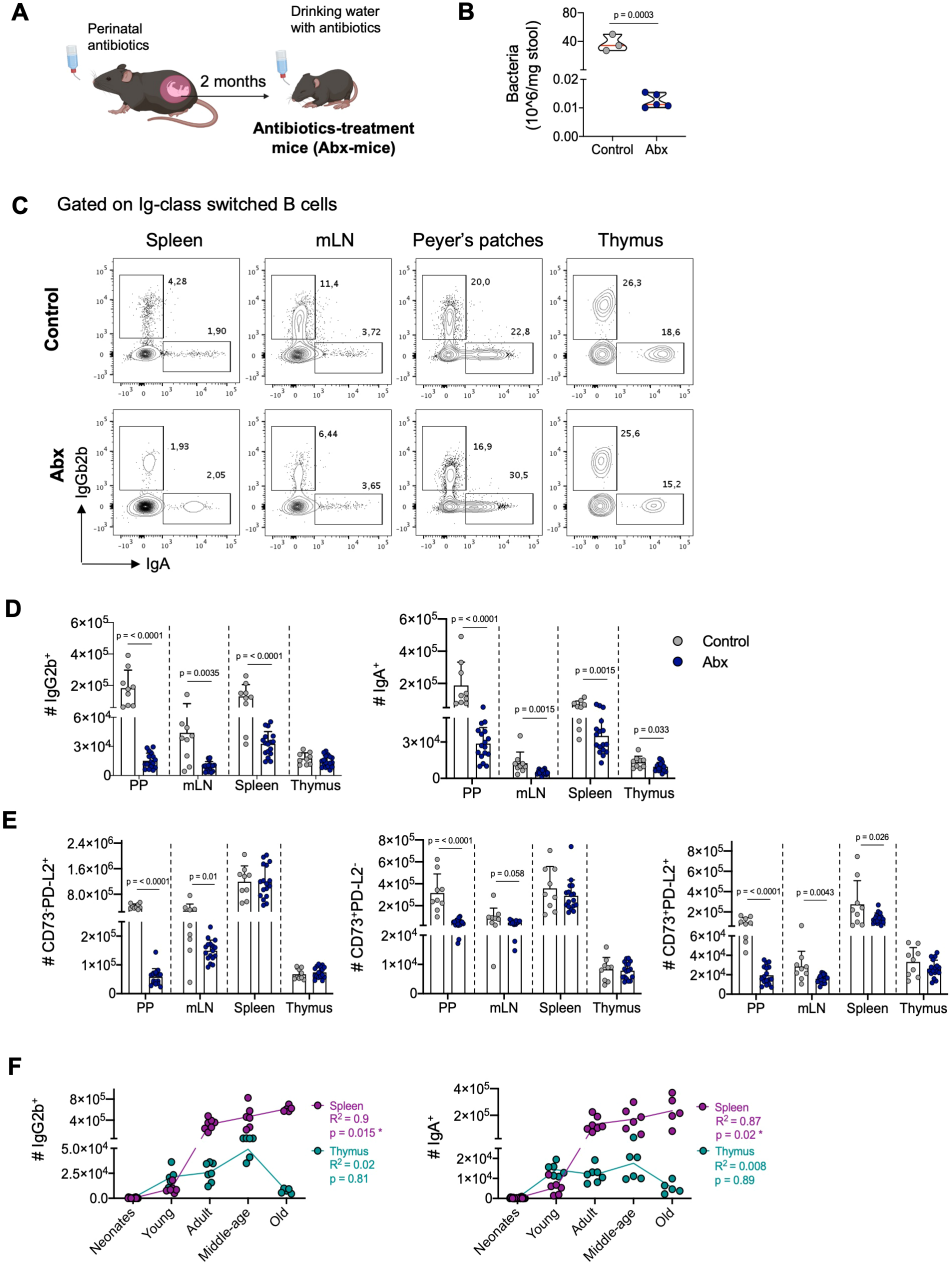
Impact of environmental antigens on thymic and peripheral memory B cells. **(A)** Schematics representation of the generation of C57BL/6 mice with reduced microbiota through perinatal antibiotics treatment. Mice were treated with antibiotics from fetal development until 8 weeks of age, at which point they were analyzed. **(B)** Quantification of fecal bacteria load by flow cytometry in the control group (n = 9) and the antibiotic-treated group (n = 17). **(C)** Representative dot plots of IgG2b^+^ and IgA^+^ B cells in the Peyer’s patches, mesenteric lymph nodes, spleen, and thymus of control (top) and antibiotic-treated (bottom) groups. **(D)** The number of IgG2b^+^ and IgA^+^ B cells in the Peyer’s patches, mesenteric lymph nodes, spleen, and thymus, as gated in **(C)**. Data are presented as mean ± standard deviation (SD). **(E)** Impact of microbiota reduction on the numbers of CD73^-^PD-L2^+^, CD73^+^PD-L2^-^, and CD73^+^PD-L2^+^ memory B cells in all analyzed organs. Data are presented as mean ± standard deviation (SD). **(F)** Correlation between the number of splenic and thymic IgG2b^+^ and IgA^+^ cells and age. Data from five age groups: neonatal mice (3-6 days old, n = 12), young (2-12 weeks old, n = 6), adults (3-9 months old, n = 7), middle-aged (10-14 months old, n = 6), and old mice (>18 months, n = 5). Each dot represents an individual mouse. Data were analyzed by unpaired t-test for comparisons **(B, D, E)**, and Spearman correlation was used to calculate goodness of fit (R^2^) and p values **(F)**. Abx, antibiotics-treatment mice; PP, Peyer’s patches; mLN, mesenteric lymph nodes.

Our observation that thymic B cells display a memory phenotype early in development, preceding peripheral memory formation, led us to hypothesize that their differentiation is independent of external stimuli, including microbiota exposure. To test this, we administered antibiotics to mice from perinatal development to reduce antigen exposure by diminishing intestinal microbiota (34–37) (**Figure 4A**). This significantly reduced the number of intestinal bacteria without affecting the body weight (**Figure 4B**, **Supplementary Figure 5A**). Analysis revealed that the frequency and number of IgG2b^+^ and IgA^+^ subsets were reduced in the spleen, mesenteric lymph nodes (mLN), and Peyer’s patches of antibiotic-treated mice. However, in the thymus, the frequency and number of IgA^+^ B cells were only slightly reduced, while the IgG2b^+^ subset showed no significant changes (**Figures 4C, D**). The effect of antibiotic treatment in the number of IgG2b^+^ and IgA^+^ cells was high in Peyer’s patches (Cohen’s d: 2.08 and 1.55, respectively), mLN (Cohen’s d: 1.44 and 1.12, respectively), and spleen (Cohen´s d: 2.21 and 1.34, respectively), whereas a considerably smaller effect was observed in the thymus (Cohen´s d: 0.48 and 0.88, respectively). Consistently, the reduction in microbiota antigens impaired the formation of memory B cells expressing CD73 and PD-L2 molecules in intestinal-associated organs, while their number in the thymus remained unaffected (**Figure 4E**). Antibiotic treatment had a very high effect in the number of CD73^-^PD-L2^+^, CD73^+^PD-L2^-^, and CD73^+^PD-L2^+^ memory B cells in Peyer’s patches (Cohen’s d: 6.13, 2.14, and 2.43, respectively) and mLN compartment (Cohen’s d: 0.95, 0.8, and 1,1, respectively), while the effect was smaller in the spleen (Cohen’s d: 0.02, 0.38 and 0.81, respectively) and thymus (Cohen’s d: 0.35, 0.13, and 0.57, respectively). These differences reflect the striking reduction in cellularity and the total number of class-switched cells in intestinal-associated organs compared to the spleen and thymus of antibiotic-treated mice (**Supplementary Figures 5B-D**). These results suggest that, unlike peripheral memory formation, thymic memory B cell differentiation is not significantly influenced by exogenous antigens.

Previous reports suggest that the progressive accumulation of memory B cells in the circulation and secondary lymphoid organs during aging results from continuous exposure to external antigens (42). To investigate whether thymic memory B cell frequency changes with age-related antigen exposure, we evaluated the number of IgG2b^+^ and IgA^+^ B cells in the spleen and thymus of mice at different ages. As expected, there was a progressive increase in IgG2b^+^ and IgA^+^ B cells in the spleen, peaking in old mice (>18 months). However, in the thymus, there was no correlation between the number of IgG2b^+^ and IgA^+^ B cells and age; instead, their number decreased in the thymus of old mice (**Figure 4F**). Similarly, memory B cells expressing CD73 and PD-L2 increased with age in the spleen but showed no correlation in the thymus (**Supplementary Figure 5F**). This indicates that thymic memory B cells do not follow the same pattern associated with aging as observed in secondary lymphoid organs.

### Critical role of cognate CD4 SP thymocytes in thymic memory B cell development

**Figure 5:**
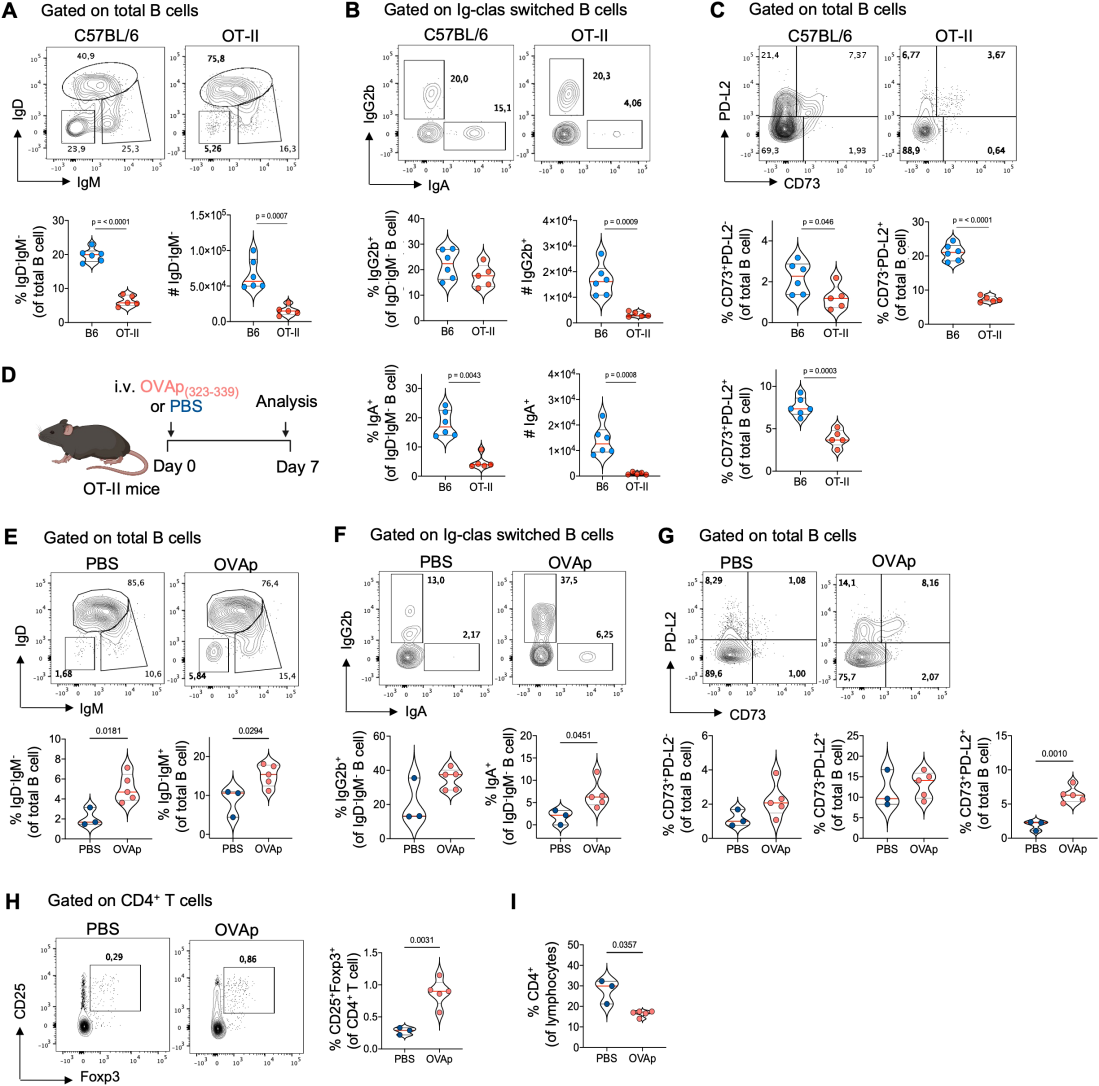
The critical role of cognate CD4 SP thymocytes interaction in thymic memory B cell development. **(A)** Representative dot plots (top), frequency, and number (bottom) of total class-switched B cell (IgD^-^IgM^-^) gated on thymic B cells from 3-month-old C57BL/6 (n = 6) and OT-II mice (n = 5). **(B)** Representative dot plots (top), frequency, and number (bottom) of IgG2b^+^ and IgA^+^ isotypes within IgD^-^IgM^-^ gated on Ig-class-switched thymic B cells from C57BL/6 (n = 6) and OT-II mice (n = 5). **(C)** Representative dot plots (top) and frequency (bottom) of antigen–specific memory B cells based on CD73 and PD-L2 expression in thymic B cells from C57BL/6 (n = 6) and OT-II mice (n = 5). **(D)** Schematics experimental representation of the administration of OVA_323-339_ peptide (OVAp) to 3-month-old OT-II mice for seven days. **(E)** Representative dot plots (top) and frequency (bottom) of total class-switched (IgD^-^IgM^-^) and IgM^+^ memory thymic B cells from PBS-injected (n = 3) and OVAp OT-II-injected mice (n = 5). **(F)** Representative dot plot (top) and frequency (bottom) of Ig class-switched IgG2b^+^ and IgA^+^ B cells within IgD^-^IgM^-^ gated on thymic B cells from PBS-injected (n = 3) and OVAp OT-II-injected mice (n = 5). **(G)** Representative dot plot (top) and frequency (bottom) of antigen–specific memory B cells based on CD73 and PD-L2 expression in thymic B cells from PBS-injected and OVAp OT-II-injected mice. **(H)** Representative dot plot (left) and frequency (right) of CD25^+^Foxp3^+^ regulatory T cells gated on CD4^+^ T cells in the thymus from PBS-injected and OVAp OT-II-injected mice. **(I)** Frequency of total CD4 SP thymocytes in PBS-injected and OVAp OT-II-injected mice. Each dot on the graph represents an individual mouse. Data were analyzed using an unpaired t-test for comparisons, except for the frequency of IgG2b^+^ B cells **(E)** and CD4^+^ T cells **(I)**, which were analyzed using the Mann-Whitney test. B6, C57BL/6; OVAp, ovalbumin peptide-injected mice.

Our previous findings suggest that thymic B cells differentiate into memory cells independently of peripheral memory formation, driven by local signals within the thymus. To investigate whether interaction with immature CD4 single-positive (SP) thymocytes drives this differentiation, we examined memory B cell subsets in OT-II mice, which have CD4^+^ T cells with a restricted T-cell receptor (TCR) repertoire specific for the non-self-antigen ovalbumin. Interestingly, OT-II mice showed a drastic reduction in total class switched B cells, IgA^+^, and IgG2b^+^ cells in the thymus (**Figures 5A, B**), without differences in the total number of thymic B cells compared to B6 mice (**Supplementary Figure 6A**). Additionally, the frequency of memory B cells expressing CD73 and PD-L2 was reduced in OT-II mice (**Figure 5C**), but not in the spleen and Peyer’s patches (**Supplementary Figures 6B, C**), indicating that the differentiation of thymic memory B cells requires cognate interaction with CD4+ T cells. This was further supported by absence of differences in thymic memory B cell subsets in OT-I transgenic mice (**Supplementary Figure 6D**).

To confirm the role of CD4 SP thymocyte interaction, we provided cognate antigen by injecting ovalbumin peptide (OVAp323-339) into adult OT-II mice (as shown in **Figure 5D**). OVAp injection increased the frequency of CD25^+^Foxp3^+^ thymic Tregs and reduced the frequency of CD4 SP thymocytes in OT-II mice (**Figures 5H, I**), indicating that the administration of OVAp was able to stimulate negative selection. Interestingly, OVAp-injected mice showed higher frequencies of total class switched, IgM^+^ memory, IgG2b^+^, and IgA^+^ B cells compared to OT-II-PBS control mice (**Figures 5E, F**). Additionally, there was an increase in memory B cells expressing CD73 and PD-L2 in the thymus, particularly in the CD73^+^PD-L2^+^ subset (**Figure 5G**). These differences were not due to an increase in cellularity or total thymic B cells in response to OVAp and were limited to the thymus, with no changes observed in the memory B cell population in the spleen and Peyer’s patches (**Supplementary Figure 7**). These results show that negative selection of CD4 SP thymocytes correlates with the differentiation thymic memory B cells and suggests that crosstalk with immature CD4+ thymocytes plays a crucial role in this process.

## Discussion

Historically, studies of memory B cells have been carried out in the context of infection or immunization. In this study, we describe the atypical differentiation of thymic resident B cells into memory cells. Many thymic B cells exhibit class-switching and display a memory phenotype under homeostasis. These characteristics are influenced by interactions with developing CD4 SP thymocytes, emerge during the neonatal stage, and occur independently of antigen exposure. In contrast, peripheral B cells follow the conventional memory B cell differentiation pathway, requiring activation through external antigen recognition.

Our findings show that many thymic B cells undergo class-switch under steady-state conditions, predominantly favoring isotypes IgG2b and IgA. These cells exhibit a unique phenotype with elevated expression of various memory and activation-associated molecules, consistent with previous studies (8, 11, 13, 43). Notably, among thymic B cells the proportion of memory B cells is higher compared to the spleen, resembling the level observed in Peyer’s patches, which are known for extensive B cell activation and differentiation triggered by intestinal antigens (40). Future studies could explore the fate of thymic memory B cells and their potential for plasma cell differentiation or re-entry into germinal center reactions, providing insight into the mechanisms governing thymic B cell dynamics.

While our study focuses on the differentiation of thymic memory B cells, other studies have also evidenced the presence of plasma cells in the thymus. Previous research from our laboratory has demonstrated the presence of IgM, IgG, and IgA antibody-secreting cells in the thymus of unimmunized mice and neonatal human samples (7, 32). Furthermore, a recent study highlights the development of plasma cells in the thymus that potently secrete IgE under homeostatic conditions (44). Interestingly, these plasma cells likely differentiate locally through an extrafollicular pathway, similar to memory B cells (44), thus the thymus appears to be capable of supporting memory B cell and plasma cell differentiation.

Recent research challenges the long-standing notion that class-switch recombination and affinity maturation occur exclusively within the GC. While this is true for somatic hypermutation, accumulating evidence suggests that class-switching can precede germinal center entry (45, 46). We identified a subset of thymic B cells expressing the GC marker GL-7, indicating a potential resemblance to activated B cells that seed germinal centers in secondary lymphoid organs. A limitation in our study is the lack of information regarding Ig repertoire and clonal composition among naïve, IgM^+^ memory and class-switched B cells. However, previous investigations utilizing Ig sequence analysis on both thymic memory B cells and plasma cells have shown an absence of somatic hypermutation, suggesting independence from the germinal center reaction (43, 44). Further supporting this notion, Cd4^cre^Bcl6^fl/fl^ transgenic mice, unable to form germinal centers, exhibit an unaffected frequency of plasma cells in the thymus, whereas a significant reduction is observed in the periphery (44). Our observation of a thymic GC-like B cell subset may correspond to locally activated cells that are precursors of memory and plasma cells, resembling the extrafollicular response without germinal center formation.

Examining the influence of reducing peripheral memory formation through perinatal antibiotic administration on the formation of memory B cells in the thymus, we observed minimal impact on thymic memory B cells, indicating their independence from peripheral B cell activation. Our findings align with a study demonstrating a comparable plasma cell number in the thymus across laboratory mice under normal conditions, germ-free mice, and mice on an antigen-free diet, with a significant reduction observed in the spleen and mesenteric lymph nodes (44). Similarly, Cukrowska reported the presence of B cells secreting IgG and IgA antibodies in the thymus of neonatal pigs, even when maintained in germ-free conditions (47). Consistently, we identified memory B that had undergone class-switch in the thymus of neonatal mice before their differentiation in secondary lymphoid organs. Similarly, Kwon demonstrated that plasma cells can be detected in the thymus of one-week-old BALB/c mice (44). These findings support our hypothesis that thymic B cells differentiated independently of peripheral B cell activation. However, the signaling mechanisms involved in these differentiation processes remain unclear. At the same time, we examined the possible association between environmental antigen exposure throughout the mice´s lifespan and memory B cell accumulation. Expectedly, an age-associated accumulation of memory subsets was observed in the spleen. In contrast, we found a decline in the number of thymic memory B cells in aged mice. Cepeda´s research also found a reduction in the frequency of class-switched IgD^-^IgM^-^ B cells in the thymus and lower expression of the transcription factor *Aire* and the *cd40* gene in mice of advanced age (8). This suggests that a potential loss of thymic B cell functionality as antigen presenting cells linked to thymic involution may be related to the reduced frequency of thymic memory B.

Given the established role of thymic B cells in central tolerance, the interaction between B cells and thymocytes during self-antigen presentation is crucial for differentiating thymic B cells into memory cells. Using the OT-II mice model, we confirmed, consistent with previous findings, a substantial reduction in Ig-class switch B cells (43). Additionally, our results indicated that all analyzed memory subsets are affected in the thymus of these mice. Interestingly, we reversed these changes by administering the OVA peptide to OT-II mice, resulting in a higher proportion of Ig-class switch and memory B cells than untreated mice. Furthermore, our data suggests an increase in the negative selection of thymocytes in OT-II mice with OVA peptide administration. This is further supported by evidence from the literature indicating a significant decrease in thymic IgD^-^IgM^-^ B cells in TCR-deficient mice and mice with B cells lacking MHC-II (43). Similarly, in CD40 or CD40L-deficient mice, thymic B cells are unable to undergo Ig-class switch, impacting other features such as increased expression of MHC-II, CD80 molecules, and the *Aire* gene (11, 43), supporting the role of B-T interaction in acquiring the characteristic phenotype of thymic B cells. The ability to engage and mediate negative selection of T cells with self-reactive specificities implies that B cell reactivity towards autoantigens would enhance the ability to interact with cognate T cells. In a study by Perera, the Ig light chains from naive and class-switched thymic B cells were cloned and expressed with the 3H9 heavy chain (predisposed to DNA reactivity) to test a potential bias in the specificity of switched B cells towards self-antigens. Interestingly, light Ig chains from switched thymic B cells generated a higher proportion of ANA reactive antibodies, suggesting that a self-reactive BCR strongly favors B-T interaction and memory B cell differentiation (43).

In conclusion, our data demonstrate that a subset of thymus-resident B cells undergo homeostatic memory B cell differentiation. This process appears to be related to their role as local thymic antigen presenting cells, requiring interaction with developing T cells, and occurs independent of peripheral B cell activation to external antigens. Nevertheless, further research is needed to elucidate other signals involved in this differentiation process and the relevance of the memory phenotype in their function.

## Data Availability

The raw data supporting the conclusions of this article will be made available by the authors, without undue reservation.

## Supplementary material

Supplementary Figure 1 Gating strategy of thymic memory B cells and intravascular staining. **(A)** Representative gating strategy of class-switched B cells and CD73^+^, PD-L2^+^, CD73^+^PD-L2^+^ memory B cell subsets. **(B)** Representative intravascular labeling of B cells in spleen, lung and thymus. 3μg of labelled anti-CD45 was intravenously injected 3 minutes prior to euthanasia, followed by cell isolation, surface staining and FACS analysis. CD45iv^+^ cells represent leukocytes present in the blood at the time organs were collected.

Supplementary Figure 2 Thymic memory B cells present a distinct phenotype profile compared to peripheral memory B cells. **(A)** Representative dot plots of IgG1^+^ and IgG2ac^+^ B cells within the IgD^-^IgM^-^ gate in the thymus of 3-month-old C57BL/6 mice (n = 3). **(B)** Percentage of IgG1^+^ and IgG2ac^+^ isotypes among IgD^-^IgM^-^ gated B cells from the spleen, Peyer´s patches, and thymus. **(C)** Principal component analysis (PCA) of experimental variables related to memory B cell subsets, applied to splenic, Peyer’s patches, and thymic B cells from 3-month-old C57BL/6 mice (n = 6). **(D-E)** t-SNE of 30.000 live B cells from spleen, Peyer’s patches, and thymus of 3-month-old C57BL/6 mice (n = 3) clustered randomly according to markers used (CD21-35, IgD, CD23, PD-L2, CD73, GL-7, and CD43). For both analyses (PCA and t-SNE), data were obtained from flow cytometry and visualized using R studio. Each dot represents an individual mouse. Data in (A) were analyzed using one-way ANOVA with Tukey’s *post-hoc* test for multiple comparisons.

Supplementary Figure 3 Thymic memory B cell formation is a general strain-independent process in mice. **(A**) Representative dot plots (left) and frequency (right) of total class-switched (IgD^-^IgM^-^) and IgM^+^ memory (IgD^-^IgM^+^) B cells within the B220^+^CD19^+^GL-7^-^ gate in the spleen, Peyer’s patches, and thymus of 3-month-old BALB/c mice (n = 4). **(B)** Representative dot plots (left) and frequency (right) of class-switched IgG2b and IgA isotypes within IgD^-^IgM^-^ gated B cells from the spleen, Peyer’s patches, and thymus of BALB/c mice (n = 4). **(C)** Dot plot of CD73 and PD-L2 expression in naïve (IgD^+^IgM^+^), IgM^+^ memory (IgD^-^IgM^+^), and Ig-class-switched (IgD^-^IgM^-^) B cells subsets in the spleen, Peyer´s patches, and thymus (n = 6). **(D)** Proportion of CD73^+^PD-L2^-^, CD73^+^PD-L2^+^, and CD73^-^PD-L2^+^ within naïve, IgM^+^ memory, and Ig-class-switched B cells gated as in (C). Each dot represents an individual mouse. Data were analyzed using one-way ANOVA with Tukey’s *post-hoc* test for multiple comparisons (A, B), except for IgM^-^IgD^-^ and IgG2b^+^ B cells, which were analyzed using the Kruskal-Wallis test with Dunn’s post-test. PP: Peyer’s patches.

Supplementary Figure 4 Circulating and resident thymic cells in parabiotic mice. **(A)** Representative dot plots (left) and frequency (right) of CD4^+^CD8^+^ thymocytes in host-derived cells in parabiotic mice (n = 6). **(B)** Dot plots (left) and frequency (right) of host-derived cells in the naïve (IgD^+^IgM^+^), IgM^+^ (IgD^-^IgM^+^), and class-switched (IgD^-^IgM^-^) B cells subsets in the spleen and thymus from parabiotic mice (n = 6). Each dot represents an individual mouse. Data were analyzed by unpaired t-test **(A)** and two-way ANOVA with Tukey’s post-test for multiple comparisons **(B).**

Supplementary Figure 5 The number of thymic memory B cells does not change in reduced microbiota mice and is not correlated with age. **(A)** Weight (g) of control (n = 9) and antibiotic-treatment mice (n = 17). **(B)** The total number (cellularity) of Peyer’s patches (PP), mesenteric lymph nodes (mLN), spleen, and the thymus from control and antibiotic-treated mice, obtained by optical microscopy. **(C)** Frequency (top) and number (bottom) of total thymic B cells (B220^+^CD19^+^) in control and antibiotic-treated mice. **(D)** Total number of Ig-class switched B cells (IgD^-^IgM^-^) in PP, mLN, spleen, and thymus from control and antibiotic-treated mice. **(E)** Correlation between the frequency and number of total B cells with age in the thymus and spleen. **(F)** Correlation between the number of CD73^+^PD-L2^+^, CD73^-^PD-L2^+^, CD73^+^PD-L2^-^ memory B cells with age in the thymus and spleen. Each dot represents an individual mouse. Data were analyzed using an unpaired t-test **(A-D)**, except for the total number of PP **(B)** and the number of splenic IgM^-^IgD^-^ B cells **(D)**, which were analyzed using the Mann-Whitney test. Pearson correlation was used to calculate goodness of fit (R^2^) and p-values **(E-F)**, except for the percentage of total B cells **(E)**, which were analyzed using the Spearman correlation. Abx, antibiotics-treatment mice; mLN, mesenteric lymph nodes.

Supplementary Figure 6 Memory B cells subsets in the spleen and Peyer´s Patches from C57BL/6 and OT-II present similar frequencies. **(A**) Frequency (top) and number (bottom) of total thymic B cells (B220^+^CD19^+^) of C57BL/6 (n = 6) and OT-II mice (n = 5). **(B)** Representative dot plots (left) and frequency (right) of total Ig-class-switched in the spleen and Peyer’s patches of C57BL/6 and OT-II mice. **(C)** Representative dot plots (left) and frequency (right) of IgG2b^+^, and IgA^+^ in the spleen and Peyer’s patches of C57BL/6 and OT-II mice. **(D)** Representative dot plots of Ig-class-switched, IgG2b^+^, IgA^+^, CD73^-^PD-L2^+^, CD73^+^PD-L2^-^, and CD73^+^PD-L2^+^ thymic B cells in OT-I mice. Data were analyzed using an unpaired t-test for comparisons, except for the frequency of thymic B cells **(A)** and IgA^+^ B cells in PP **(C)**, which were analyzed using the Mann-Whitney test. B6: C57BL/6.

Supplementary Figure 7 Ovalbumin-peptide does not affect the number of memory B cells in the spleen and Peyer´s Patches. **(A)** Total cell number (cellularity) of thymus in PBS-injected (n = 3) and OVAp-injected OT-II mice (n = 5). **(B)** Frequency (left) and number (right) of total thymic B cells (B220^+^CD19^+^) in PBS-injected and OVAp-injected OT-II mice. **(C)** Representative dot plots (left) and frequency (right) of Ig-class-switched and IgM^+^ memory B cells in the spleen and Peyer’s patches in PBS– and OVAp-injected OT-II mice. **(D)** Representative dot plots (top) and frequency (bottom) of IgG2b^+^ and IgA^+^ B cells in the spleen and Peyer’s patches in PBS– and OVAp-injected OT-II mice. All the data were analyzed using an unpaired t-test for comparisons. OVAp: ovalbumin peptide-injected mice.
