# Correction: Early-life homeostatic differentiation of thymus-resident B cells into memory B cells

**DOI:** 10.3389/fimmu.2026.1783042

**Published:** 2026-01-26

**Authors:** Justine Castañeda, Lilian Poblete, Mariana V. Rosemblatt, Daniela Sauma, Mario Rosemblatt, María Rosa Bono, Sarah Nuñez

**Affiliations:** 1 Escuela de Postgrado, Facultad de Medicina, Universidad de Chile, Santiago, Chile; 2 Departamento de Biología, Facultad de Ciencias, Universidad de Chile, Santiago, Chile; 3 Facultad de Medicina y Ciencia, Universidad San Sebastián, Sede Los Leones, Santiago, Chile; 4 Centro Ciencia & Vida, Santiago, Chile

**Keywords:** thymic B cells, memory B cell differentiation, Ig class-switching, T cell development, negative selection

Author María Rosa Bono was erroneously omitted as corresponding author. The correct corresponding authors are Sarah Nuñez and María Rosa Bono.

The original version of this article has been updated.

Author María Rosa Bono was erroneously omitted as equal contributing corresponding author.

The original version of this article has been updated.

