# Supplementary figures and images for: Early-life homeostatic differentiation of thymus-resident B cells into memory B cells
Source: Front Immunol. 2025 Mar 28;16:1567788. doi: 10.3389/fimmu.2025.1567788 (PMC11985456; doi:10.3389/fimmu.2025.1567788)

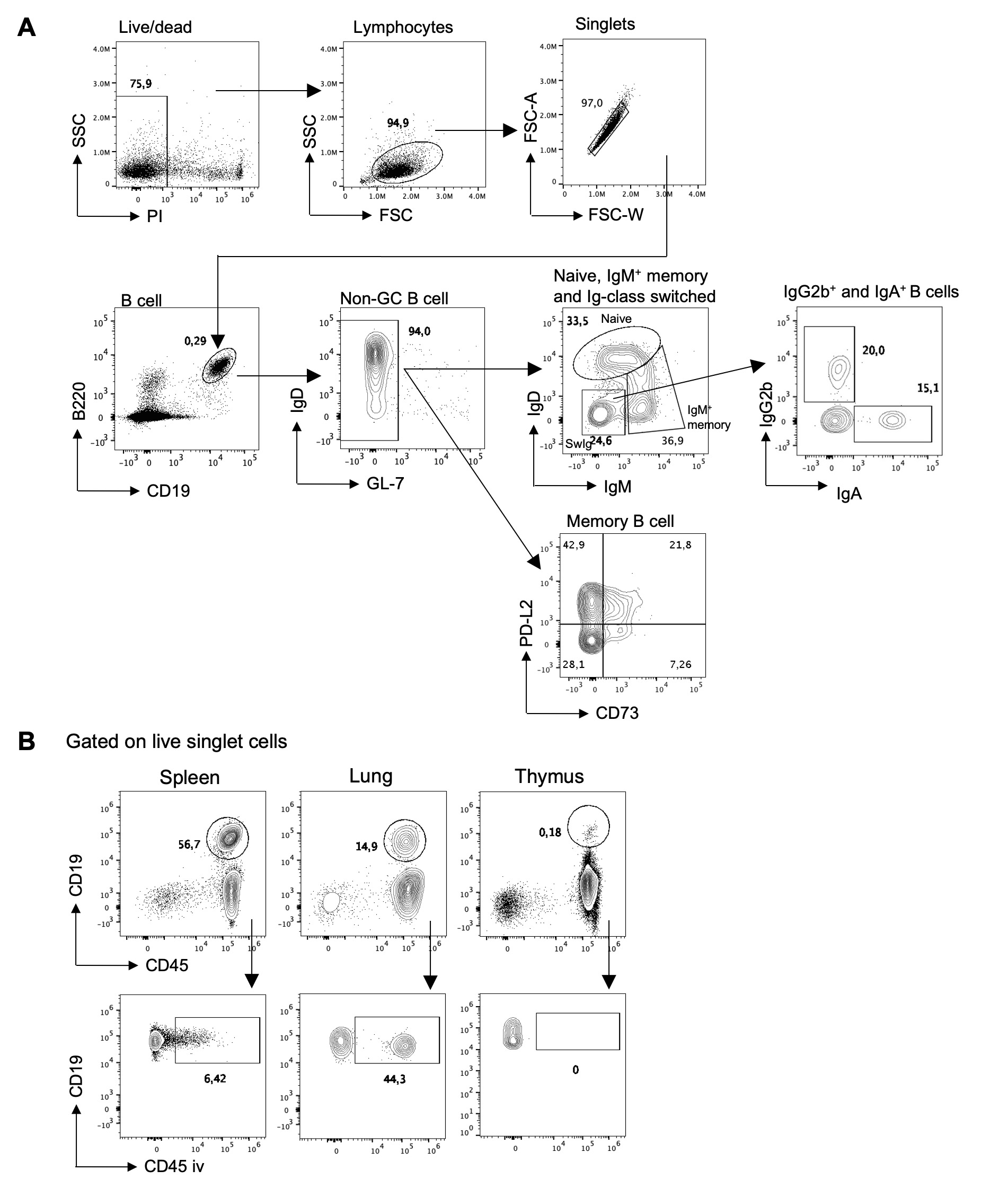

Supplement: Supplementary Figure 1 — Gating strategy of thymic memory B cells and intravascular staining. (A) Representative gating strategy of class-switched B cells and CD73+, PD-L2+, CD73+PD-L2+ memory B cell subsets. (B) Representative intravascular labeling of B cells in spleen, lung and thymus. 3μg of labelled anti-CD45 was intravenously injected 3 minutes prior to euthanasia, followed by cell isolation, surface staining and FACS analysis. CD45iv+ cells represent leukocytes present in the blood at the time organs were collected. [file Image1.jpeg]

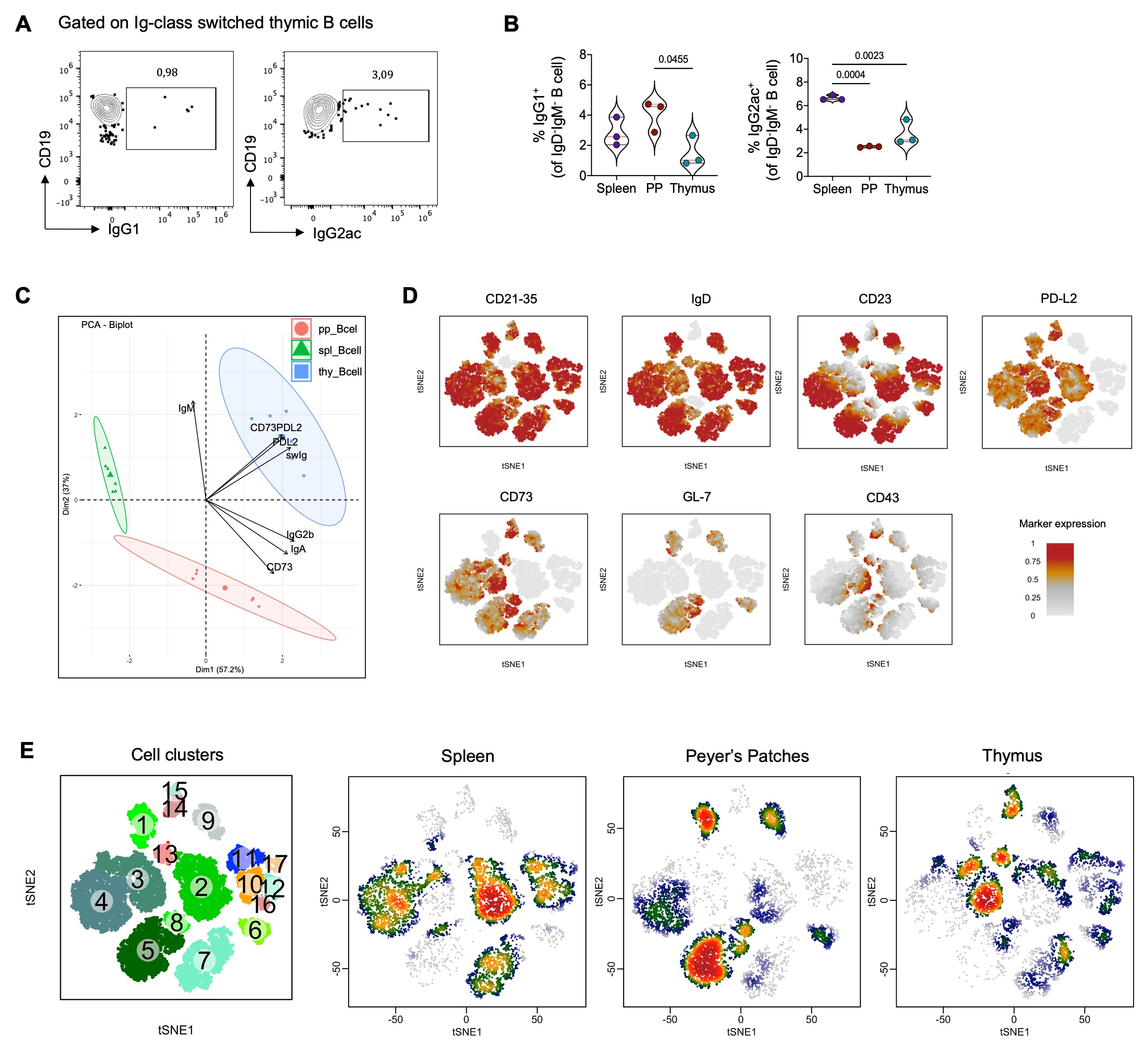

Supplement: Supplementary Figure 2 — Thymic memory B cells present a distinct phenotype profile compared to peripheral memory B cells. (A) Representative dot plots of IgG1+ and IgG2ac+ B cells within the IgD-IgM- gate in the thymus of 3-month-old C57BL/6 mice (n = 3). (B) Percentage of IgG1+ and IgG2ac+ isotypes among IgD-IgM- gated B cells from the spleen, Peyer´s patches, and thymus. (C) Principal component analysis (PCA) of experimental variables related to memory B cell subsets, applied to splenic, Peyer’s patches, and thymic B cells from 3-month-old C57BL/6 mice (n = 6). (D-E) t-SNE of 30.000 live B cells from spleen, Peyer’s patches, and thymus of 3-month-old C57BL/6 mice (n = 3) clustered randomly according to markers used (CD21-35, IgD, CD23, PD-L2, CD73, GL-7, and CD43). For both analyses (PCA and t-SNE), data were obtained from flow cytometry and visualized using R studio. Each dot represents an individual mouse. Data in (A) were analyzed using one-way ANOVA with Tukey’s post-hoc test for multiple comparisons. [file Image2.jpeg]

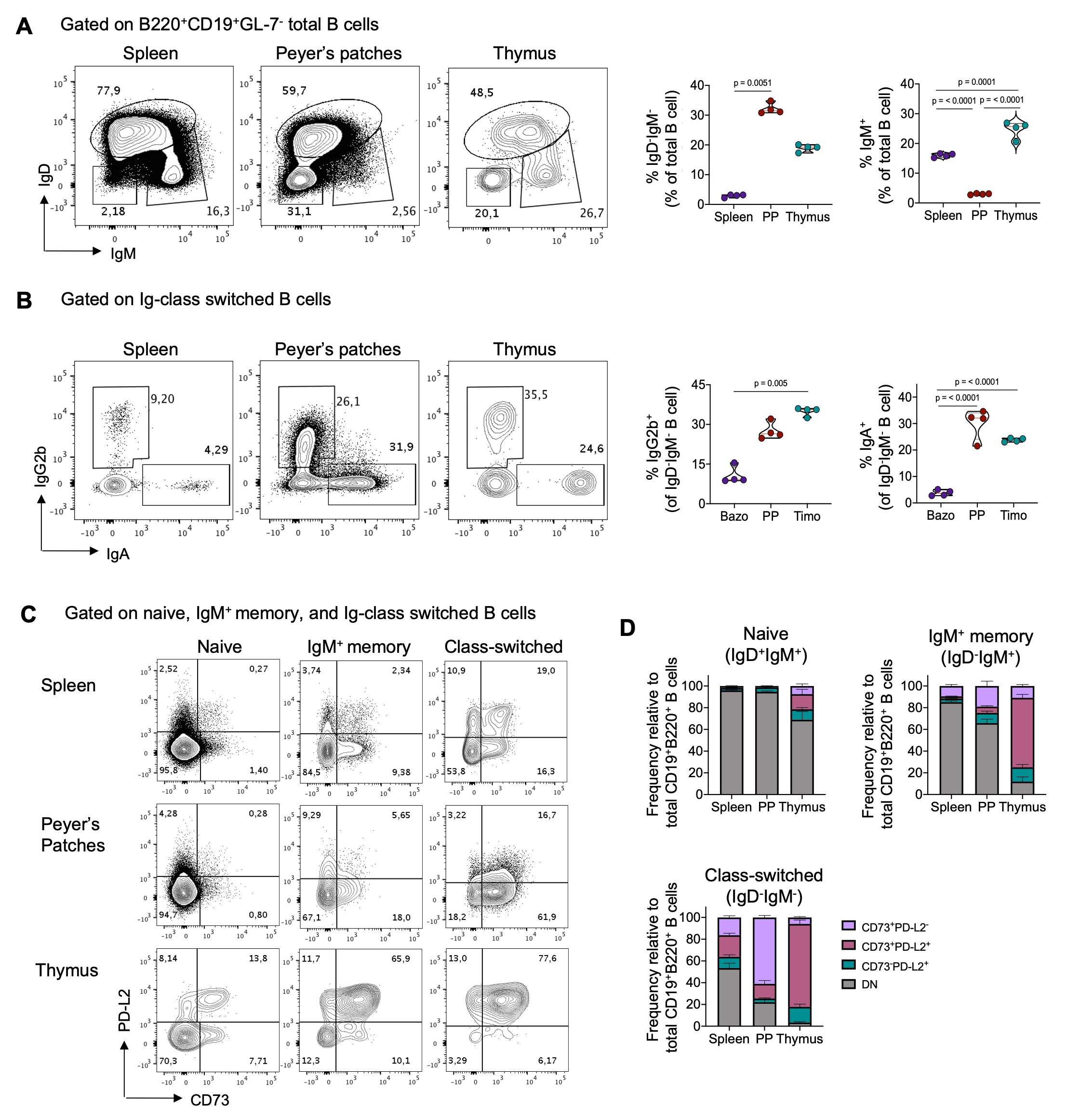

Supplement: Supplementary Figure 3 — Thymic memory B cell formation is a general strain-independent process in mice. (A) Representative dot plots (left) and frequency (right) of total class-switched (IgD-IgM-) and IgM+ memory (IgD-IgM+) B cells within the B220+CD19+GL-7- gate in the spleen, Peyer’s patches, and thymus of 3-month-old BALB/c mice (n = 4). (B) Representative dot plots (left) and frequency (right) of class-switched IgG2b and IgA isotypes within IgD-IgM- gated B cells from the spleen, Peyer’s patches, and thymus of BALB/c mice (n = 4). (C) Dot plot of CD73 and PD-L2 expression in naïve (IgD+IgM+), IgM+ memory (IgD-IgM+), and Ig-class-switched (IgD-IgM-) B cells subsets in the spleen, Peyer´s patches, and thymus (n = 6). (D) Proportion of CD73+PD-L2-, CD73+PD-L2+, and CD73-PD-L2+ within naïve, IgM+ memory, and Ig-class-switched B cells gated as in (C). Each dot represents an individual mouse. Data were analyzed using one-way ANOVA with Tukey’s post-hoc test for multiple comparisons (A, B), except for IgM-IgD- and IgG2b+ B cells, which were analyzed using the Kruskal-Wallis test with Dunn’s post-test. PP: Peyer’s patches. [file Image3.jpeg]

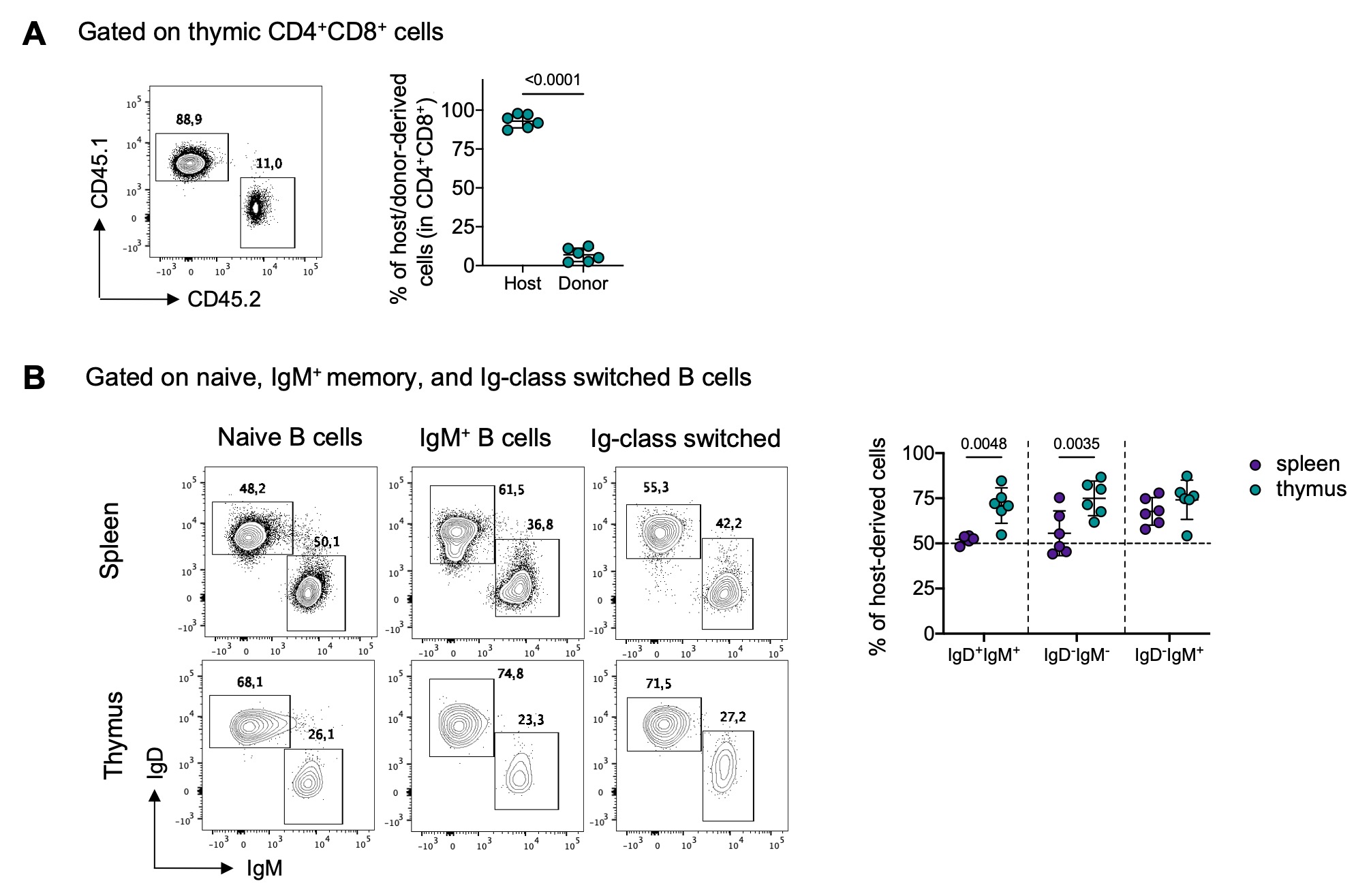

Supplement: Supplementary Figure 4 — Circulating and resident thymic cells in parabiotic mice. (A) Representative dot plots (left) and frequency (right) of CD4+CD8+ thymocytes in host-derived cells in parabiotic mice (n = 6). (B) Dot plots (left) and frequency (right) of host-derived cells in the naïve (IgD+IgM+), IgM+ (IgD-IgM+), and class-switched (IgD-IgM-) B cells subsets in the spleen and thymus from parabiotic mice (n = 6). Each dot represents an individual mouse. Data were analyzed by unpaired t-test (A) and two-way ANOVA with Tukey’s post-test for multiple comparisons (B). [file Image4.jpeg]

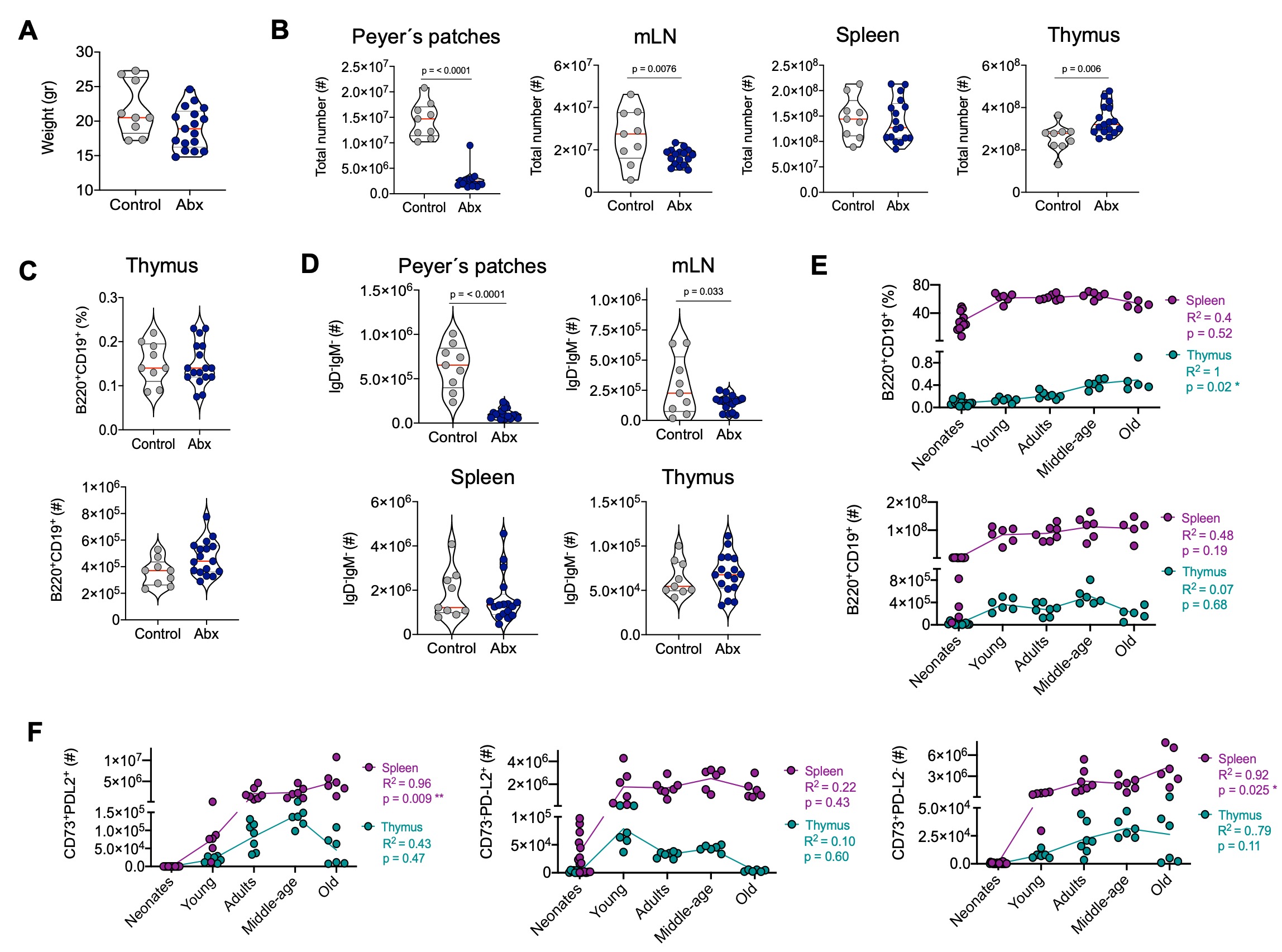

Supplement: Supplementary Figure 5 — The number of thymic memory B cells does not change in reduced microbiota mice and is not correlated with age. (A) Weight (g) of control (n = 9) and antibiotic-treatment mice (n = 17). (B) The total number (cellularity) of Peyer’s patches (PP), mesenteric lymph nodes (mLN), spleen, and the thymus from control and antibiotic-treated mice, obtained by optical microscopy. (C) Frequency (top) and number (bottom) of total thymic B cells (B220+CD19+) in control and antibiotic-treated mice. (D) Total number of Ig-class switched B cells (IgD-IgM-) in PP, mLN, spleen, and thymus from control and antibiotic-treated mice. (E) Correlation between the frequency and number of total B cells with age in the thymus and spleen. (F) Correlation between the number of CD73+PD-L2+, CD73-PD-L2+, CD73+PD-L2- memory B cells with age in the thymus and spleen. Each dot represents an individual mouse. Data were analyzed using an unpaired t-test (A-D), except for the total number of PP (B) and the number of splenic IgM-IgD- B cells (D), which were analyzed using the Mann-Whitney test. Pearson correlation was used to calculate goodness of fit (R2) and p-values (E-F), except for the percentage of total B cells (E), which were analyzed using the Spearman correlation. Abx, antibiotics-treatment mice; mLN, mesenteric lymph nodes. [file Image5.jpeg]

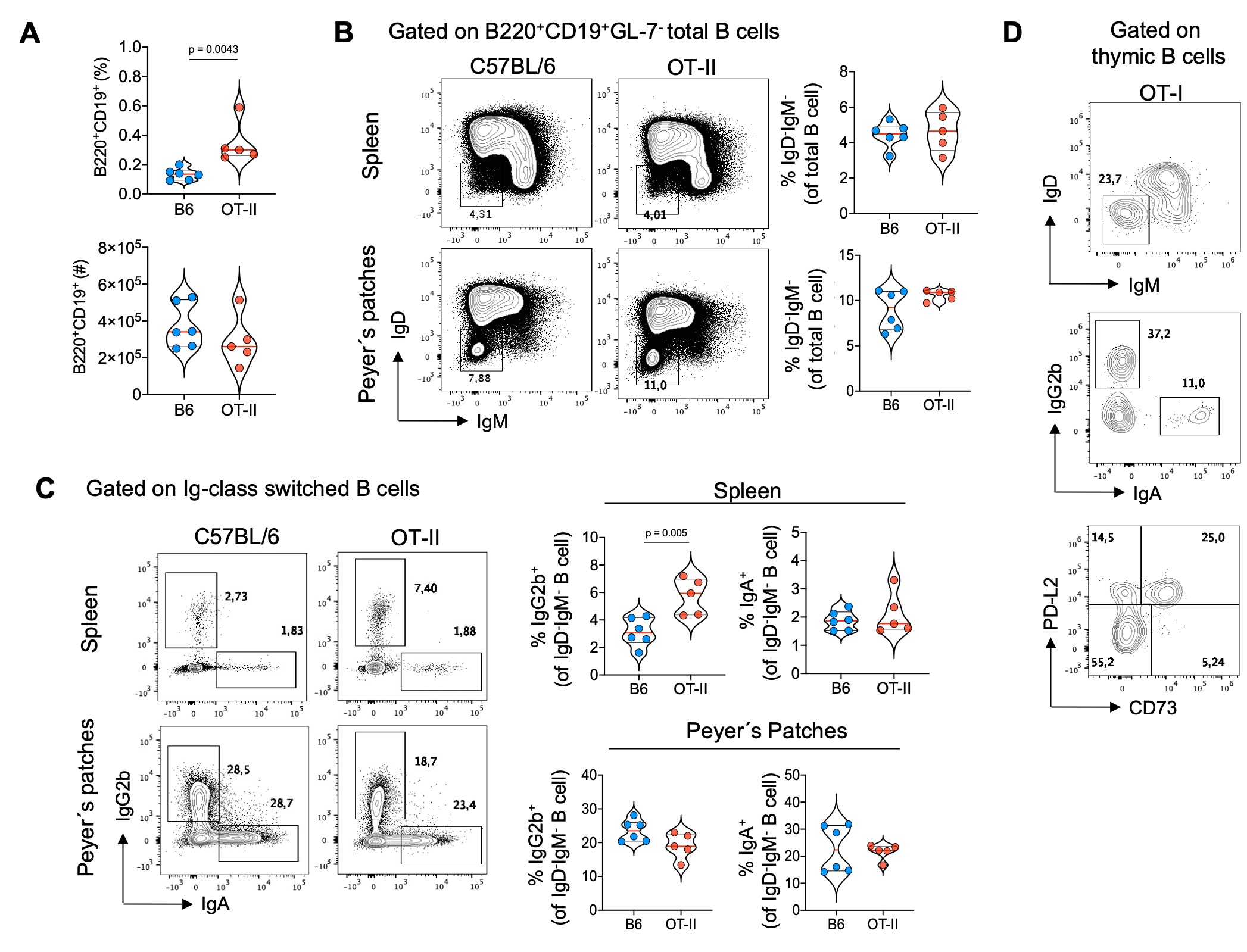

Supplement: Supplementary Figure 6 — Memory B cells subsets in the spleen and Peyer´s Patches from C57BL/6 and OT-II present similar frequencies. (A) Frequency (top) and number (bottom) of total thymic B cells (B220+CD19+) of C57BL/6 (n = 6) and OT-II mice (n = 5). (B) Representative dot plots (left) and frequency (right) of total Ig-class-switched in the spleen and Peyer’s patches of C57BL/6 and OT-II mice. (C) Representative dot plots (left) and frequency (right) of IgG2b+, and IgA+ in the spleen and Peyer’s patches of C57BL/6 and OT-II mice. (D) Representative dot plots of Ig-class-switched, IgG2b+, IgA+, CD73-PD-L2+, CD73+PD-L2-, and CD73+PD-L2+ thymic B cells in OT-I mice. Data were analyzed using an unpaired t-test for comparisons, except for the frequency of thymic B cells (A) and IgA+ B cells in PP (C), which were analyzed using the Mann-Whitney test. B6: C57BL/6. [file Image6.jpeg]

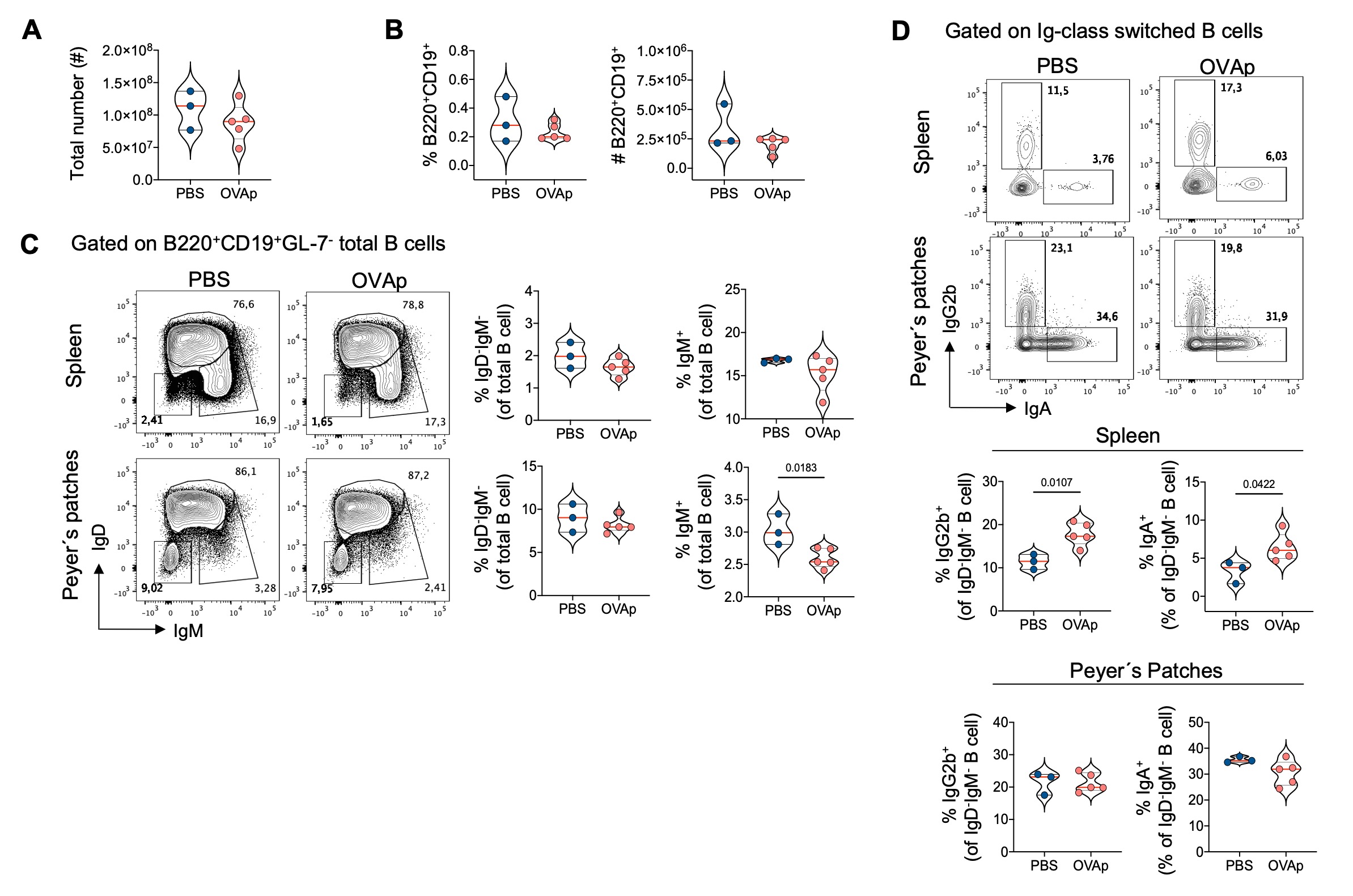

Supplement: Supplementary Figure 7 — Ovalbumin-peptide does not affect the number of memory B cells in the spleen and Peyer´s Patches. (A) Total cell number (cellularity) of thymus in PBS-injected (n = 3) and OVAp-injected OT-II mice (n = 5). (B) Frequency (left) and number (right) of total thymic B cells (B220+CD19+) in PBS-injected and OVAp-injected OT-II mice. (C) Representative dot plots (left) and frequency (right) of Ig-class-switched and IgM+ memory B cells in the spleen and Peyer’s patches in PBS– and OVAp-injected OT-II mice. (D) Representative dot plots (top) and frequency (bottom) of IgG2b+ and IgA+ B cells in the spleen and Peyer’s patches in PBS– and OVAp-injected OT-II mice. All the data were analyzed using an unpaired t-test for comparisons. OVAp: ovalbumin peptide-injected mice. [file Image7.jpeg]
